# Prevalence of Diabetes Mellitus and Impaired Fasting Glucose, Associated with Risk Factors in Rural Kazakh Adults in Xinjiang, China

**DOI:** 10.3390/ijerph120100554

**Published:** 2015-01-09

**Authors:** Shugang Li, Shuxia Guo, Fei He, Mei Zhang, Jia He, Yizhong Yan, Yusong Ding, Jingyu Zhang, Jiaming Liu, Heng Guo, Shangzhi Xu, Rulin Ma

**Affiliations:** Department of Preventive Medicine, School of Medicine, Shihezi University, Beier Road, Shihezi City, Xinjiang Uyghur Autonomous Region 832000, China; E-Mails: lishugang@ymail.com (S.L.); hefei71@126.com (F.H.); zmberry@foxmail.com (M.Z.); hejia123.shihezi@163.com (J.H.); erniu19880215@sina.com (Y.Y.); 13399931625@163.com (Y.D.); yfyxxzjy@126.com (J.Z.); liujiaming@shzu.edu.cn (J.L.); guoheng@shzu.edu.cn (H.G.); shzuxushangzhi@126.com (S.X.); marulin@126.com (R.M.)

**Keywords:** diabetes mellitus, impaired fasting glucose, rural, associated factors

## Abstract

*Objective*: This study aimed to estimate the prevalence of diabetes mellitus (DM) and impaired fasting glucose (IFG) in a Kazakh population aged ≥18 years living in the YiLi District of Xinjiang, China and to evaluate the associated risk factors of diabetes. *Methods*: Randomly selected adults, living for at least 6 months in the YiLi District in Xinjiang had their clinical characteristics and standard blood chemistries measured. DM and IFG were defined according to WHO 1999 criteria. The adjusted odds ratio (ORs) and 95% confidence intervals were calculated for the association of diabetes risk factors in multivariate logistic regression models. *Results*: A total of 3919 subjects were randomly selected. The age-and gender-standardized prevalence of DM and IFG were 5.9% and 10.0%, respectively. The prevalence of DM and IFG increased with age and BMI. Prevalence of 7.4%, 12.2% in males and 4.9%, 8.6% in females for DM and IFG. Compared by sex, prevalence of DM and IFG was higher in males. Prevalence of 3.4%, 8.1% in normal, 6.7%, 11.9% in overweight and 12.0%, 13.0% in obesity for diabetes and IFG. In the multivariable logistic models, male sex, older age, unmarried, overweight, obesity, hypertension, triglycerides and smoking were all significantly associated with an increased risk of diabetes. *Conclusions*: The prevalence of DM and IFG among minorities was lower than the overall national level both in men and women (9.7% in total, 10.6% in males, 8.8% in females), and also lower than among the Han ethnicity (9.26%) which predominates in China today.

## 1. Introduction

Diabetes mellitus (DM) is rapidly becoming one of the most common non-communicable diseases globally [1]. In 2013 there were 382 million people with diabetes, and this number is expected to rise to 592 million by 2035. Population growth, aging of population, and urbanization with associated lifestyle change is likely to lead to a 55% increase in worldwide numbers with diabetes by 2035. China has become the country with the largest number of people with diabetes in the World [2].

In a cross-sectional study in 2000–2001 involving a nationally representative sample of 15,540 adults, 35 to 74 years of age, the prevalence of diabetes and impaired fasting glucose (IFG) were 5.5% and 7.3%, respectively [3]. A recent nationwide diabetes epidemiological study indicated that prevalence of DM and pre-diabetes has increased to 9.7% and 15.5%, translating into 92.4 million adults with diabetes [4]. In China, a rapid increase in the prevalence of DM has been reported [3,4]. China currently has a population of 1.34 billion [5], therefore the potential effect of diabetes on the global and national economy and society are very large. However, with China’s economic growth, population aging, nutritional transition and urbanization, especially in economically underdeveloped areas such as Xinjiang, there have been few studies of the pattern of the DM epidemic among the Han nationality and its major risk factors, especially since no studies have included the Xinjiang minority epidemic of DM and IFG in a rural area. To address this gap in knowledge, we conducted a study aimed to estimate the prevalence of DM and IFG and to evaluate the associated risk factors of diabetes in the Kazakh adult population.

## 2. Subjects and Methods

### 2.1. Subjects and Sampling

The survey was conducted from 2009 to 2010. A multistage (region-county-township-village) stratified cluster random sampling method was used to select participants. At the beginning, we chose one representative region (Yili) according to the geographical distribution of the population in Xinjiang (which is located in the northwest region in China). After that, we randomly selected one county (Xinyuan County) from the eight counties in Yili. Then, one township (Nalati Township) was randomly chosen from six townships in Xinyuan County. In the last stage, a stratified sampling method was used to draw corresponding villages (six in Nalati) in one township. With informed consent, we interviewed permanent residents (residing for more than 6 months in the village) of Kazakh nationality aged 18 and above for investigation.

A total of 4400 people were selected and invited to participate in the survey. A total of 3919 people (1552 men and 2367 women) completed the study. The overall response rate was 89.1%. Fasting serum glucose values were obtained from 3919 adults. Informed consent was obtained from each participant prior to data collection.

### 2.2. Data Collection

Data collection included questionnaire interviews, clinical measurements and venous blood collection. Data collection was all conducted by face-to-face interview in their homes. All of the investigators underwent strict training in the methodology and principles of the study programmer as well as in the necessary skills for the study prior to the start of the study. The investigators included professional Kazak language translators.

### 2.3. Questionnaire Interviews

A standard questionnaire was administered by trained investigators to obtain information on demographic characteristics, anthropometry, personal and family medical history, lifestyle risk factors and educational, social details including occupation, educational status and physical activity levels. The questionnaire was based on Chinese survey, however, the investigator who were Kazakh and could speak Kazakh and Chinese, were well trained to control the quality of survey. In addition, the investigators were nurses who worked in the hospital in YiLi District and therefore professionals who could collect accurate information from the interviewees.

### 2.4. Clinical Measurements

Blood pressure, body weight, height, and waist circumference were measured by standard methods. Height was measured to the nearest centimeter using a tape measure attached to the wall and with the subject standing as erect as possible. Body weight was measured to use a digital bathroom scale, with the subjects barefoot and wearing lightweight clothing. Waist circumference (at the level of the navel) and hip circumference were measured in duplicate with the subjects standing and at the end of expiration while breathing normally, and the average of the two values used in the study analyses. Body mass index (BMI) was calculated as the ratio of weight to height squared (kg/m^2^). Participants with a BMI ≥ 25 kg/m^2^ and < 28 kg/m^2^ were classified as overweight, and those with a BMI ≥ 28 kg/m^2^ were classified as obese (World Health Organization (WHO) 1997 standards) [6].

Systolic blood pressure (SBP) and diastolic blood pressure (DBP) were measured three times in each subject, using a mercury sphygmobolometer in the sitting position after a 15 min rest, and the values then averaged. Hypertension was defined as systolic blood pressure (SBP) ≥ 140 mm·Hg, or diastolic blood pressure (DBP) ≥ 90 mm·Hg, or current use of any antihypertensive medication within 2 weeks or any combination of the above.

### 2.5. Blood lipid and Glucose Tests

Venous blood was collected into 3 mL anti-coagulation tubes and another 3 mL tube without anti-coagulation agent tube and subjected immediately to centrifugal separation of plasma and serum, then stored in a −80 °C refrigerator. Fasting plasma glucose(FPG) and serum lipid profiles, including total cholesterol(TC), triglycerides(TG), HDL-cholesterol and LDL-cholesterol, were also obtained using an automated biochemical analysis instrument (OLYMPUS AU2700, Tokyo, Japan).

### 2.6. Diagnostic Criteria for Diabetes

Participants without a prior diagnosis of diabetes were categorized according to the 1999 World Health Organization diagnostic criteria were used to diagnose diabetes as follows: undiagnosed diabetes (FPG ≥ 7.0 mmol/L) and IFG (FPG: 6.1–6.9 mmol/L) [7].

### 2.7. Statistical Analysis

SPSS software (version 16.0 for Windows; SPSS, Chicago, IL, USA) was used for the statistical analyses. Descriptive data were expressed as means ± standard deviations (SD) and analyzed using *t*-test. Categorical variables were expressed as numbers or percentages and analyzed using the Chi-square test. A multinomial logistic-regression analysis was used to determine the adjusted odds ratios (ORs) and 95% confidence intervals (CIs) of the independent predictors of diabetes. All tests for statistical significance were two-sided and a *p* value of < 0.05 was considered statistically significant.

## 3. Results

### 3.1. Characteristics of the Study Subjects

Among the 3919 surveyed subjects, 1552 (39.60%) were male and 2367 (60.40%) were female. There were significant differences in age between males and females, with mean ages of 45.09 ± 13.53, 43.59 ± 13.02 years (Table 1). 

**Table 1:** Characteristics of the study population and prevalence of cardiovascular risk factors.

Characteristics	Total (*N* = 3919)	Males (*N* = 1552)	Females (*N* = 2367)	*χ*^2^*/t*	*p value*
Age (years)	44.18 ± 13.24	45.09 ± 13.53	43.59 ± 13.02	3.460	<0.01
Weight(kg)	64.80 ± 12.93	70.90 ± 12.72	60.79 ± 11.41	25.34	<0.01
Height (cm)	163.04 ± 8.74	169.88 ± 6.95	158.55 ± 6.63	50.806	<0.01
Body mass index (kg/m^2^)	24.35 ± 4.61	24.56 ± 4.13	24.21 ± 4.89	2.350	<0.05
Waist circumference (cm)	85.72 ± 12.30	88.425 ± 12.12	83.94 ± 12.10	11.345	<0.01
Hip circumference (cm)	98.10 ± 9.16	98.75 ± 8.38	97.67 ± 9.62	3.728	<0.01
Systolic blood pressure(mm Hg)	128.7 ± 23.87	131.84 ± 22.95	126.64 ± 24.25	6.706	<0.01
Diastolic blood pressure (mm Hg)	82.94 ± 14.55	84.6 ± 14.15	81.85 ± 14.70	5.810	<0.01
Fasting Blood-glucose (m mol/L)	5.30 ± 1.36	5.40 ± 1.57	5.24 ± 1.20	3.440	<0.01
Total Cholesterol (m mol/L)	4.55 ± 1.32	4.53 ± 12.23	4.56 ± 1.38	−0.677	0.488
Triglycerides (m·mol/l)	1.25 ± 1.01	1.39 ± 1.18	1.16 ± 0.87	6.700	<0.01
LDL-Cholesterol (m·mol/L)	2.40 ± 0.87	2.46 ± 0.91	2.36 ± 0.84	3.568	<0.01
HDL-Cholesterol (m·mol/L)	1.41 ± 0.62	1.35 ± 0.57	1.44 ± 0.65	−4.987	<0.01
Overweight (%)	27.4	28.6	26.6	1.870	0.171
Obesity (%)	18.2	18.4	18.1	0.750	0.784
Hypertension (%)	36.7	42.0	33.3	30.968	<0.01
Dyslipidemia (%)	49.5	53.7	46.8	18.212	<0.01

Data are presented as means ± SD or proportions (%).

Table 1 shows the characteristics of the study population (means ± SD) and prevalence of cardiovascular risk factor (%) for sex. Descriptive data were expressed as means ± standard deviations (SD) and analyzed using *t*-test (Male *vs*. Female, *p* < 0.01). Categorical variables were expressed as numbers or percentages and analyzed using the Chi-square test (Male *vs*. Female, *p* < 0.01). Compared by sex, the average levels for weight and height, SBP, DBP, and prevalence of hypertension and hyperglycemia were higher among males, while females had significantly higher HDL-cholesterol levels than did the males. However, there were no significant differences between males and females in total cholesterol, overweight and obesity.

### 3.2. Prevalence of DM and IFG in Different Ages and Sex

**Figure 1:**
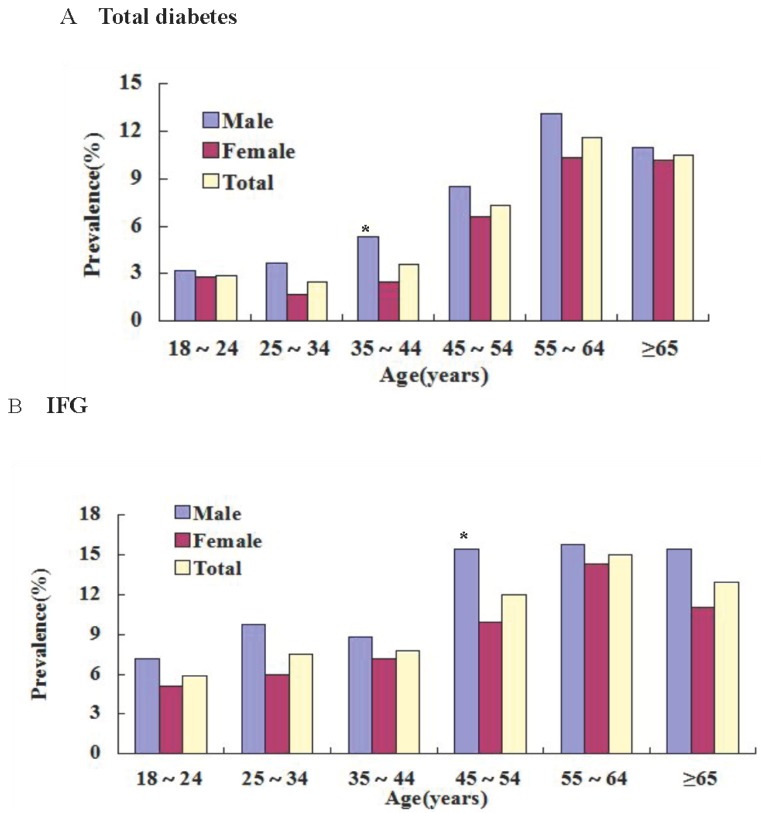
Shows age-specific prevalence of diabetes and impaired fasting glucose (IFG) among Kazakh adults 10 years of age or older. The prevalence of total diabetes (**Panel A**) and predicaments (**Panel B**) among men and women are shown, according to age. * Male *vs*. Female, *p* < 0.05 with chi-square test.

The age-and gender-standardized prevalence was 5.9% for diabetes and 10.0% for IFG (Table 2). In both males and females, the prevalence of DM and IFG increased with age (*p* < 0.05) (Figure 1). In 55–64 age group, prevalence of DM and IFG was the highest in both males and females (Figure 1). Prevalence of 7.4%, 12.2% in males and 4.9%, 8.6% in females for DM and IFG. Compared by sex, prevalence of DM and IFG was higher in males (*p* < 0.05). In 35–44 age group, prevalence of DM was higher in males (*p* < 0.05). In 45–54 age group, prevalence of IFG was higher in males (*p* < 0.05) (Table 2 and Figure 1). Table 2 shows the gender- and age-specific prevalence rates of Diabetes mellitus (DM) and impaired fasting glucose (IFG) in the Kazakh population aged ≥ 18 years in Xinjiang. 

**Table 2:** Prevalence of diabetes and impaired fasting glucose (IFG) by gender in Kazakh (n(%)).

Age Group (Years)	Total Group				Male				Female				
	Diabetes		IFG		Diabetes		IFG		Diabetes		IFG	
	*N*	%	*N*	%	*N*	%	*N*	%	*N*	%	*N*	%
18–24	10	2.9	20	5.9	4	3.2	9	7.2	6	2.8	11	5.1
25–34	19	2.5	57	7.5	11	3.7	29	9.7	8	1.7	28	6.0
35–44	36	3.6	78	7.8	19	5.3	32	8.8	17	2.5 *	46	7.2
45–54	72	7.3	118	12.0	32	8.5	58	15.4	40	6.6	60	9.9 *
55–64	72	11.6	93	15.0	39	13.1	47	15.8	33	10.3	46	14.3
≥65	22	10.5	27	12.9	10	11.0	14	15.4	12	10.2	13	11.0
Total prevalence	231	5.9	393	10.0	115	7.4	189	12.2	116	4.9 *	204	8.6 *

For age and gender. * Male *vs*. Female, *p* < 0.05.

The diagnostic criteria for DM and IFG was the the 1999 World Health Organization diagnostic criteria DM (FPG ≥ 7.0 mmol/L), IFG (FPG: 6.1–6.9 mmol/L). Categorical variables were expressed as numbers or percentages and analyzed using the Chi-square test (Male *vs*. Female, *p* < 0.05). 

### 3.3. Prevalence of DM and IFG in Different BMI

**Figure 2:**
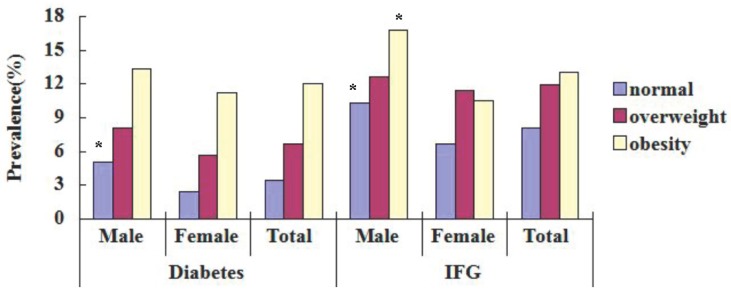
Prevalence of diabetes and fasting glucose (IFG) with different BMI in the Kazakh population in Xinjiang aged ≥ 18 years. * Male *vs.* Female, *p* < 0.05 with chi-square test.

Prevalence of 3.4%, 8.1% in normal, 6.7%, 11.9% in overweight and 12.0%, 13.0% in obesity for diabetes and IFG (Table 3). In both males and females, the prevalence of DM and IFG increased with BMI (*p* < 0.05) (Figure 2). In both males and females, prevalence of DM and IFG was the highest in the obesity group and lowest in the normal one (Figure 2). Compared by sex, prevalence of DM in normal was higher in males, prevalence of IFG in obesity and normal was higher in males (*p* < 0.05) (Table 3, Figure 2). Table 3 shows prevalence of diabetes and impaired fasting glucose (IFG) in different BMI for Kazakhs aged ≥ 18 years in Xinjiang. Body mass index (BMI) was calculated as the ratio of weight to height squared (kg/m^2^). Participants with a BMI ≥ 18 kg/m^2^ and < 25 kg/m^2^ as normal, a BMI ≥ 25 kg/m^2^ and < 28 kg/m^2^ were classified as overweight, and those with a BMI ≥ 28 kg/m^2^ were classified as obesity (World Health Organization (WHO) 1997 standards) [6]. Categorical variables were expressed as numbers or percentages and analyzed using the Chi-square test (Male *vs.* Female, *p* < 0.01). 

**Table 3:** Prevalence of diabetes and impaired fasting glucose (IFG) in different BMI for Kazak (n(%)).

BMI	Total Group				Male				Female			
	Diabetes		IFG		Diabetes		IFG		Diabetes		IFG	
	n	%	n	%	n	%	n	%	n	%	n	%
Normal	73	3.4	172	8.1	41	5.0	85	10.3	32	2.4 *	87	6.7 *
Overweight	72	6.7	128	11.9	36	8.1	56	12.6	36	5.7	72	11.4
Obesity	86	12.0	93	13.0	38	13.3	48	16.8	48	11.2	45	10.5 *
Total	231	5.9	393	10.0	115	7.4	189	12.2	116	4.9 *	204	8.6 *

* Male *vs.* Female, *p* < 0.05.

### 3.4. Adjusted ORs of Associated Factors for Diabetes

The results of multivariate logistic-regression analyses showed that diabetes were statistically significantly and positively associated with sex, age, overweight, obesity, hypertension, triglycerides, and smoking (Table 4).

**Table 4:** Multivariate adjusted odds ratios (95% CI) for factors predictive of diabetes.

Explanatory Variables	β	Odds Ratio	OR 95%CI	*p* *value*
Females sex (*vs.* males)	−0.355	0.702	0.52–0.946	0.020
Age(control:18–24 year)				
25–34	0.286	1.332	0.592–2.994	0.488
35–44	0.47	1.600	0.744–3.437	0.229
45–54	1.088	2.969	1.441–6.119	0.003
55–64	1.422	4.144	1.995–8.610	<0.01
≥65	1.421	4.140	1.746–9.816	0.001
unmarried (*vs.* married)	1.552	4.723	3.382–6.596	<0.01
Overweight	1.153	3.166	1.119–8.958	0.030
Obesity	0.375	1.454	1.04–2.035	0.029
Hypertension	0.481	1.618	1.183–2.213	0.003
Triglycerides	0.773	2.167	1.574–2.983	<0.01
Smoking (*vs*. no smoking)	0.521	1.684	1.199–2.366	0.003

Table 4 shows the multivariate adjusted odds ratios (95% CI) for factors predictive of diabetes among Kazakh adults according to logistic-regression analyses. Finally, we calculated ORs and 95% CIs of diabetes in a multiple logistic regression model adjusted for age and gender. A multinomial logistic-regression analysis, using a forward stepwise method, was used to determine the adjusted odds ratios (ORs) of the independent predictors of diabetes. A *p* value of < 0.05 was considered statistically significant. 

## 4. Discussion

In the present study, we found a very high prevalence of DM and IFG in the Kazakh adult population (5.9% and 10.0%, respectively). Prevalence of 7.4%, 12.2% in males and 4.9%, 8.6% in females for DM and IFG. In both males and females, the prevalence of DM and IFG increased with age (*p* < 0.05), the prevalence of DM and IFG increased with BMI (*p* < 0.05). Compared by sex, prevalence of DM and IFG was higher in males.

The results of the present study reveal that the standardized prevalence of DM among Kazakh adult population living in Xinjiang is 5.9% (7.4% in males and 4.9% in females). This rate is lower than the 2007–2008 China National Diabetes and Metabolic Disorders Study level (9.7% in total, 10.6% in males and 8.8% in females) [4], the prevalence among individuals in Shanghai (12.6%) [8], in Qingdao (6.1%) [9], and is also lower than the prevalence of DM in the Chinese population of Taiwan (9.0%) [10], while the prevalence in some Chinese provinces such as in Hunan (1.5%) [11], Guizhou (1.9%) [11], Guangxi (2.5%) [11] and Hubei (2.7%) [12] was still relatively low. In general, the prevalence among minorities was lower than the overall national level both in men and women [4], and also lower than in the Han ethnicity which predominates in China today [8,9,10], but was higher than for other ethnicities in other regions of China [11,12], but lower than for the Uygur (6.23%) and Han (9.26%) in Xinjiang [13], and lower in the ethnic minority [14] and Manchu, Korean ethnicities group of China [15]. Diabetes level differences among men than women and the literature are consistent [4,8,13]. The overall prevalence of diabetes in the US was estimated to be 12.6% in 2005–2006 [16]. The prevalence of DM in the Amsterdam (The Netherlands) population was significantly higher in in the Turkish (5.6%) and Moroccan (8.0%) ones, compared to our study [17]. These data show that ethnic minorities living in Western countries may have a higher prevalence of diabetes.

All this data shows that there are differences between different ethnic groups, regions and countries in the prevention of DM. The differences in the prevalence of DM in these populations may be due to differences in the environment and lifestyle, or to the different ethnicity of the populations in the different studies. For example, what is notable different in the Chinese Han nationality in the dietary structure.

The results of the present study reveal that 9.3% of Kazakh adults had IFG, an important risk factor in the development of diabetes. This rate is higher than the reported prevalence of IFG in the Kazakh population of Fuhai County of Xinjiang Province (6.94%) [18], and the prevalence among Uyghur populations in the Hetian region of Xinjiang (4.77%) [19]. It is almost three times higher than the standardized prevalence of IFG among the adult population living in Guangzhou (3.3%) [20] This suggests that many of those with IFG in this population may become diabetic in future if their IFG remains undiagnosed and/or uncontrolled. This means that screening needs to be conducted regularly to identify those individuals with IFG and to provide them with effective interventional measures to prevent the development of diabetes in later life.

Furthermore, the present study shows that there is generally significant difference in the prevalence of DM and IFG between men and women. Kazakh men had a higher prevalence of DM and IFG compared with women. In the 55–64 age group, the prevalence of DM and IFG was the highest. The prevalence rates of diabetes and impaired glucose tolerance of the Chinese populations rise in older age groups [22]. The Kazakhs are a traditional nomad population of the northern regions of China. However, with the economic and technological development of Xinjiang Province, the traditional customs have changed, and part of young and male labor force probably went to work in the city, while the females became responsible for all the housework and animal grazing work. The males are thus less active compared with the females, so the higher DM and IFG prevalence of men may be due to their less activities. The Kazak population generally has a lower educational level, which may make Kazakh males and females have lower awareness about diabetes and health management. Furthermore, compared with other cities, there is lower awareness of diabetes in the rural population in Xinjiang, especially among the Xinjiang ethnic minorities.

This study investigated the major Kazakh dietary patterns, which mainly involve the consumption of beef, mutton, pasta, butter, cheese and other milk products, but less intake of vegetables and fruits. Pasta contains high amounts of carbohydrates, but beef and mutton are rich in saturated fatty acids. This provides sufficient materials for the synthesis of fat in the body, resulting in lipid metabolism disorders. This study shows that lipid metabolism disorders are a risk factor for DM, and affect glucose metabolism. Therefore, the special Kazakh dietary patterns and hereditary factors may cause the higher prevalence of DM and IFG.

In the multivariable logistic models, male sex, older age, unmarried, overweight, obesity, hypertension, triglycerides and smoking were all significantly associated with an increased risk of diabetes (Table 3). Furthermore, these results indicate that metabolic disorders such as overweight (27.40%), obesity (18.22%), hypertension (36.72%) and dyslipidemia (49.53%) are more prevalent than ever, and are higher than among the Han nationality and ethnic minorities in other areas [23,24], and are all strongly associated with both diabetes and IFG. In this study, in both men and women, the prevalence of DM and IFG increased with BMI (*p* < 0.05). Prevalence was 3.43%, 8.07% in normal, 6.70%, 11.92% in overweight and 12.04%, 13.03% in obesity for diabetes and IFG. Our data support the notion that overweight and obesity in particular are strong risk factors for type 2 diabetes, a finding that is consistent with those reported previously in various racial/ethnic populations [22]. However, No smoking seems to be a protective factor against diabetes. Kazakh men smoke less or are almost non-smokings, which is a national characteristic, which is very different from the Han nationality. These findings also support that preventive lifestyle interventions should be targeted at lowering both overweight and obesity levels in the Chinese Kazakh population. In tandem with economic development and changes toward a lifestyle that lacks physical activity and is rich in high-fat diet, the prevalence of diabetes of the Chinese populations are on the rise [22]. Therefore, it is essential that an integrated health-education programme be established in communities to both boost public awareness of diabetes, its risk factors and complications, and promote healthy habits in citizens, including having regular health examinations to identify early-stage diabetes and early treatment interventions to delay the development of diabetes and reduce diabetes-related complications.

Several limitations of the current study merit consideration. First, dietary intake and physical activity were assessed in our study, but more data is missing, and dietary intake and physical activity are not in the final regression equation. Second, we study a Xinjiang Province minority, which differs from the Chinese Han nationality in their diet and living habits. Third, to ensure comparability across studies, we used the World Health Organization criteria to define DM and IFG in our study. Different criteria in the study may produce different results. Fourthly, personal economic factors were not included in this study, and if we can study the relationship between the economic conditions and the prevalence of DM, the findings may be different in various kinds of races/ethnic groups.

## 5. Conclusions

In summary, the prevalence of DM and IFG among the rural adult Kazakh population of Xinjiang Province has increased considerably over the recent decades, with the number of pre-diabetics growing. This population-based study characterized baseline risk factors for DM among Chinese Kazakh adults in Xinjiang and provides a biological resource that enables further investigation of numerous hypotheses related to genetic exposures to account for the differences between ethnic minorities and Han Chinese that have both undergone rapid socioeconomic transformations in recent years. Our initial analysis identifies several important risk factors (e.g., overweight and obesity) that affect the high prevalence of IFG and diabetes and be useful for the development of effective strategies for DM prevention among the Chinese Kazakh population.
